# Promoter-proximal transcription factor binding is transcriptionally active when coupled with nucleosome repositioning in immediate vicinity

**DOI:** 10.1093/nar/gku596

**Published:** 2014-07-31

**Authors:** Vinod Kumar Yadav, Ram Krishna Thakur, Bruce Eckloff, Aradhita Baral, Ankita Singh, Rashi Halder, Akinchan Kumar, Mohammad Parwez Alam, Tapas K. Kundu, Raj Pandita, Tej K. Pandita, Eric D. Wieben, Shantanu Chowdhury

**Affiliations:** 1 GNR Center for Genome Informatics, Institute of Genomics and Integrative Biology, Delhi, India; 2 Proteomics and Structural Biology Unit, Institute of Genomics and Integrative Biology, Delhi, India; 3 Advanced Genomics Technology Center, Mayo Clinic, Rochester, MN, USA; 4 Dr B.R. Ambedkar Centre for Biomedical Research, University of Delhi, Delhi 110 007, India; 5 Transcription and Disease Laboratory, Molecular Biology and Genetics Unit, Jawaharlal Nehru Centre for Advanced Scientific Research, Jakkur P.O., Bangalore 560064, India; 6 Department of Radiation Oncology, The Houston Methodist Research Institute, Houston, TX 77030, USA

## Abstract

Previous studies have analyzed patterns of transcription, transcription factor (TF) binding or mapped nucleosome occupancy across the genome. These suggest that the three aspects are genetically connected but the cause and effect relationships are still unknown. For example, physiologic TF binding studies involve many TFs, consequently, it is difficult to assign nucleosome reorganization to the binding site occupancy of any particular TF. Therefore, several aspects remain unclear: does TF binding influence nucleosome (re)organizations locally or impact the chromatin landscape at a more global level; are all or only a fraction of TF binding a result of reorganization in nucleosome occupancy and do all TF binding and associated changes in nucleosome occupancy result in altered gene expression? With these in mind, following characterization of two states (before and after induction of a single TF of choice) we determined: (i) genomic binding sites of the TF, (ii) promoter nucleosome occupancy and (iii) transcriptome profiles. Results demonstrated that promoter-proximal TF binding influenced expression of the target gene when it was coupled to nucleosome repositioning at or close to its binding site in most cases. In contrast, only in few cases change in target gene expression was found when TF binding occurred without local nucleosome reorganization.

## INTRODUCTION

Although transcription factors (TFs) can recognize cognate binding sites on the nucleosomal surface, functional binding is almost always associated with chromatin modification, remodeling and finally displacement or compaction of nucleosomes. Specific epigenetic signals determine the site and state of this reorganization based on the bound TF, leading to cellular differentiation. Modification of the cellular phenotype from a group or ‘community’-like (epithelial) state to a more solitary (mesenchymal) form (epithelial to mesenchymal transition, EMT) is a basic developmental feature that has been well studied (reviewed in (1,2)). Metastasis or spreading of cancer from the site of origin is also manifested with enhanced mesenchymal features in tumor cells (3,4). In this context, though gene expression networks for the EMT are well studied, regulation of the nucleosomal or chromatin state is poorly understood (5).

Though there are several minor variations, the overall chromatin architecture of a given transcription unit may be summarized as: a ‘−1’ nucleosome positioned upstream of the transcription start site (TSS), a nucleosome-free region (NFR) followed by a ‘+1’ nucleosome downstream of TSS, in addition to an array of positioned nucleosomes throughout the gene body (6). This basic organization is stabilized by modified histones or histone variants placed at specific regions within genes (7,8). Organizational change in chromatin such as altered positioning of the +1 and/or −1 nucleosome with resultant change in the NFR (9), altered inter-nucleosomal spacing due to chromatin compaction (10–12) and/or histone variant occupancy near TSS (13) may result in transcriptional response (14–16). Together these can modulate accessibility of genomic DNA *in vivo* toward binding of TFs (6). This model is supported by high-resolution nucleosome maps generated for human promoters by subjecting chromatin to micrococcal nuclease (MNase) and detecting the undigested DNA with tiling arrays (17) or deep sequencing (MNase-Seq) (15).

By employing the yeast system, nucleosome dynamics during transcriptional changes has been investigated in detail. It was found that nucleosome reorganization as a result of physiological perturbations by means of heat-shock induced large-scale transcriptome changes in yeast. However, at the nucleosomal level, most changes were limited to one or two nucleosomes per promoter (16). Furthermore, genome-wide analysis of nucleosome dynamics during meiotic development in yeast revealed dramatic reorganization of chromatin (9). A combined analysis of nucleosome architecture, transcriptional states and status of DNA binding factors from publicly available data sets in yeast suggested DNA binding factors may control promoter nucleosome architecture (18). Other studies found that nucleosome repositioning events may facilitate TF binding and gene expression upon androgen treatment (19), during differentiation of hematopoietic stem cells to erythrocytes (20), or interferon-beta activation following virus infection (21). Together, these suggest a process where nucleosome remodelers, general TFs and the transcriptional elongation machinery together orchestrate the nucleosome-positioning pattern *in vivo* (22–24).

This understanding prompts further interesting questions regarding both the nature of TF/nucleosome interactions and to what extent this influences transcriptional response. For example, in earlier studies, induced physiological perturbations activated multiple TFs (19–21), it is not clear how unique site-specific TF binding influences nucleosome (re)organizations locally (in close vicinity of their binding sites). Another pertinent question is: do all sites occupied by a TF, and associated nucleosome occupancy changes result in altered gene expression? Keeping these in mind, we sought to study binding of single TF that would also induce the physiological change. A candidate TF was first identified from analysis of early and advanced lung cancer transcriptome profiles. *In vivo* binding sites of the TF, nucleosome positions and transcriptome profiles of both the metastatic and induced non-metastatic state in lung cancer cells were then determined (see Scheme S1 in Supplementary Information for a summary of the overall design) and using these, the correlation between target site occupancy, nucleosome reorganization and their combined effect on the transcriptome was examined. Results that suggest a model where TF binding coupled with nucleosome reorganizations that influence transcription are: (i) almost always associated with nucleosome repositioning that is at close proximity to the TF binding site (TFBS) and (ii) constrained to specific loci, and not spread over the whole genome.

## MATERIALS AND METHODS

### Chromatin immunoprecipitation and sequencing

Chromatin immunoprecipitation (ChIP) assays were performed following the Fast ChIP protocol (25). Briefly, two plates were made with 1 × 10^5^ A549 cells in each, on achieving 80% confluency the first plate was transfected with the clone pcDNA3-NME2-Myc to induce non-metastatic 2 (NME2). The second plate was left untreated (un-induced condition). After 48 h, ChIP was performed using cells from each plate independently and cells were fixed with 1% formaldehyde for 10 min, lysed and sheared (∼300 bp) using a Misonix 3000 sonicator. Twenty-five per cent of lysate was used to isolate input chromatin using phenol–chloroform and ethanol precipitation. The remaining lysate was pre-cleared using protein-A sepharose beads and further divided into two equal portions: one part was immunoprecipitated using antibody specific to the Myc epitope (clone 9E10, anti-*c*-Myc monoclonal, Sigma) and a negative control of immunoprecipitation was prepared by adding isotypic control (IgG) to the second portion. Immunoprecipitation was done by incubating overnight at 4°C with 2 μg of antibody. The un-induced experiment was treated in a similar fashion before immunoprecipitation with nm23-h2-L-16 antibody (sc-17587, Santa Cruz Biotechnology Inc.); IgG was used as the respective isotypic control. Immune complexes were collected using herring sperm DNA-saturated protein-A Sepharose, washed and eluted using Chelex-100 after proteinase K treatment. Reads were aligned (mapped) to the unmasked human reference genome (NCBI v36, hg18) using the MAQ (Mapping and Assembly with Qualities) (26) after clipping to 24 bp based on quality scores. The sequences were then mapped to the reference human genome (NCBI Build 36, UCSC hg18). Only those that aligned to the reference genome were further considered for peak generation. To accommodate variations relative to the reference genome, up to two mismatches were allowed. Commercially available small interfering RNAs (siRNAs) from Dharmacon, Inc. USA, were used to silence expression of NME2 (transient depletion) in A549 wherever required.

### Peak generation following ChIP-seq

The resulting sequence read distribution was processed with ChIP-Seq peak locator utility, CisGenome (26). CisGenome uses a conditional binomial model to identify regions in which the ChIP reads are significantly enriched relative to the control reads. A false discovery rate of 10% was considered while predicting NME2 target regions. In order to filter out low-quality sites we applied two post-processing options boundary refinement and single-strand filtering.

### Mononucleosome preparation and hybridization to promoter tiling array

A549 cells (NME2-induced or -depleted, along with respective control cells) were grown in T-75 flasks till 80% confluency in Dulbecco's modified Eagle's medium, trypsinized, pelleted and washed with ice-cold 1X phosphate buffered saline. Five times the packed pellet volume of 1X hypotonic buffer was added, cells homogenized, IGEPAL (a non-ionic, non-denaturing detergent) was added to final concentration of 0.6% lysate, vortexed and centrifuged at 12 000 revolutions per minute. Pellet (nuclei) was resuspended in MNase digestion buffer and incubated on ice. OD (Optical Density) was measured and 1 unit of MNase/OD was added to the suspension. Following incubation at 37ºC for 30 min the reaction was stopped using stop-buffer, treated with proteinase K (1 mg/ml) overnight at 37ºC before phenol–chloroform purification and ethanol precipitation to pellet down the mononucleosomal DNA. Precipitated DNA was washed with 70% alcohol and dissolved in water. DNase-treated cells were taken as control. Mixture containing 3 μg of DNA, 50 mMTris-Cl, 5 mM MgCl_2_ and 0.3 μg/μl of random hexamers was incubated at 95ºC for 5 min and chilled to 4ºC. 5 μl dNTP (1.2 mM dGTP, dCTP, dATP and 0.25 mM dUTP) and 50 units of Klenow were incubated first at 22ºC for 10 min, then 37ºC for 30 min and finally 95ºC for 5 min and chilled as above. Fifty units of Klenow were added again and the mix first kept at 22ºC for 10 min and then 37ºC for 30 min. Reaction was stopped by phenol–chloroform followed by purification using Qiagen columns. Purified product was fragmented and labeled as per Affymetrix Chromatin Immunoprecipitation Assay protocol before hybridization to Gene Chip Human Promoter Tiling array 1.0R (Affymetrix) as per manufacturer's protocol. The control DNase-treated cells were also subjected to labeling reactions as above before hybridization. Nucleosome positions were identified using iChIP (Bioconductor package).

### Nucleosome occupancy analysis

A549 cells depleted for NME2 were generated using commercially available short hairpin RNAs (from Origene Inc., USA; catalog no. TR311160) and stable cell clones were selected in presence of puromycin. For analysis of nucleosome repositioning, we considered a distance of 300 bp (+/− 150 bases), in other words, a nucleosome was denoted as repositioned in the NME2-induced condition when detected beyond 300 bp of a nucleosome found in the cells before NME2 induction. In order to avoid arbitrary assignments, in cases where a gene belonged to more than one category (while assigning NME2 nucleosome associations during repositioning analysis (Figure 5)) it was considered in all the respective cases.

**Figure 1.**
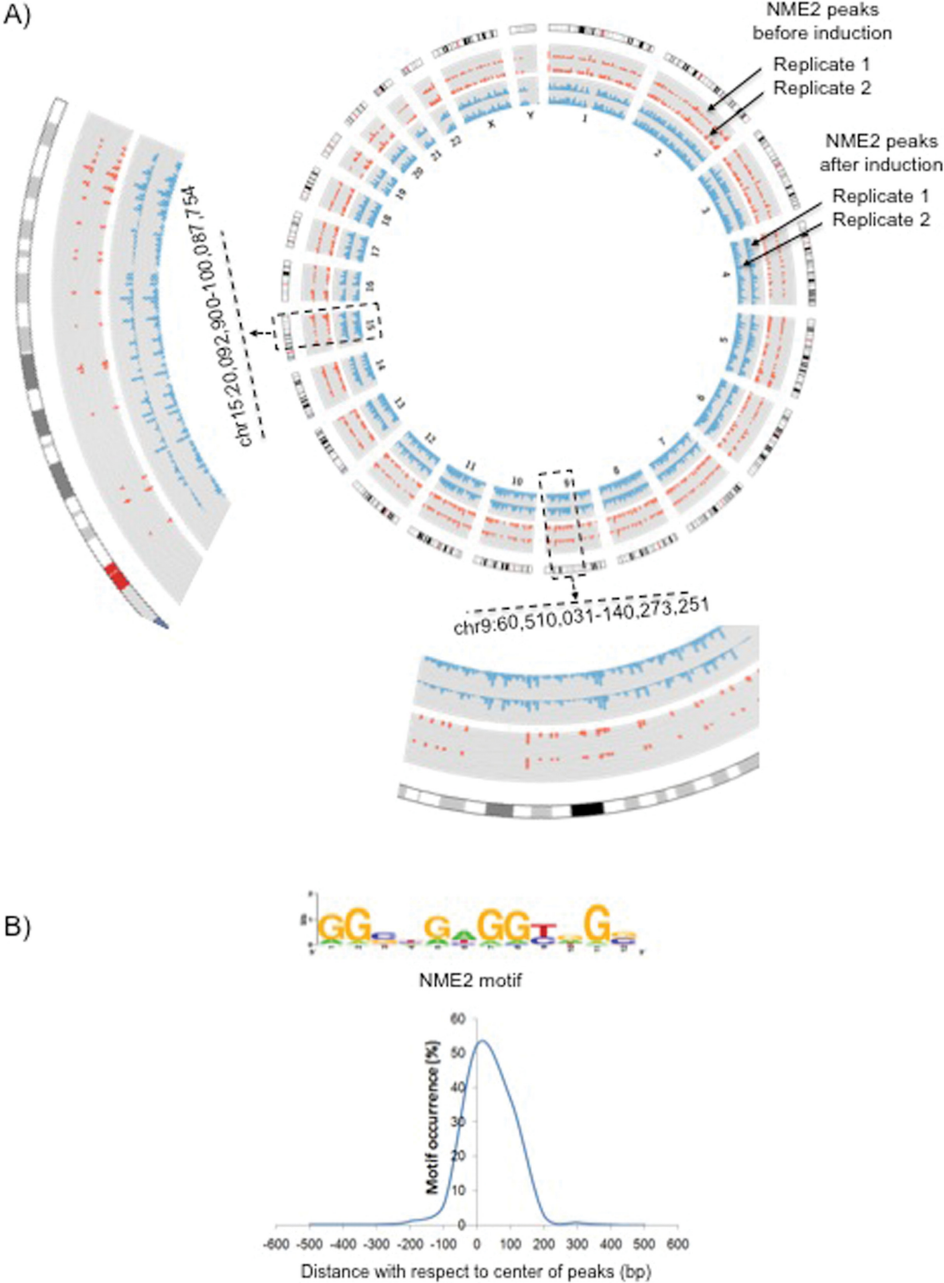
NME2 binding sites before or after inducing NME2. (**A**) A Circos plot showing ChIP-seq peak distribution of NME2 on all chromosomes (1–22, and X and Y); peaks in red represent NME2 binding sites before induction; peaks in blue represent NME2 binding sites after induction (replicates are shown in both cases). Two projections from chromosomes 9 and 15 are shown. (**B**) Twelve-mer consensus NME2 binding motif identified using Gibbs sampler (upper panel)—distribution of the 12-mer motif within NME2 peaks constructed from read counts is shown in the lower panel.

**Figure 2.**
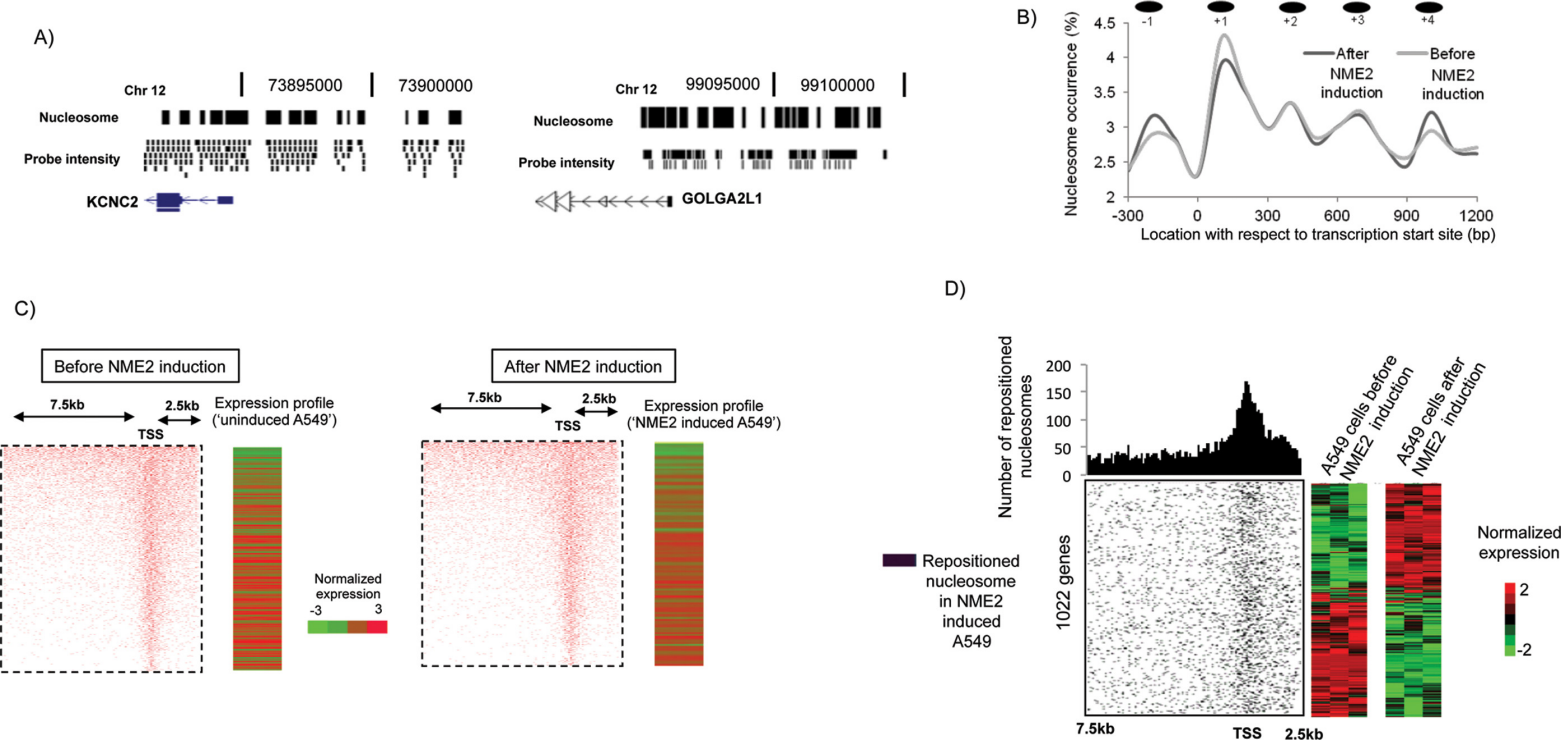
Nucleosome positions detected in A549 cells before and after inducing NME2. (**A**) UCSC browser representation of nucleosome positions with corresponding probe intensity at two loci on chromosome 12. (**B**) Nucleosome occupancy around TSS and expression level of corresponding genes in cells before (left panels) and after NME2 induction (right panels); gene expression was normalized within respective cases by z-transformation with respect to the mean expression level of the data sets. (**C**) Frequency of occurrence and location of nucleosomes around TSS. −1, +1, +2, +3, +4 denote sequential presence of nucleosomes with respect to their occurrence from TSS; percentage of total number of nucleosomes found in respective cases, before or after induction of NME2. (**D**) Left panel: distribution of repositioned nucleosomes around TSSs in NME2-induced cells relative to the status in cells before NME2 was induced; number of repositioned nucleosomes in a window size of 100 bp is shown on top. Right panel: expression of corresponding genes shown in triplicate before and after NME2 induction.

**Figure 3.**
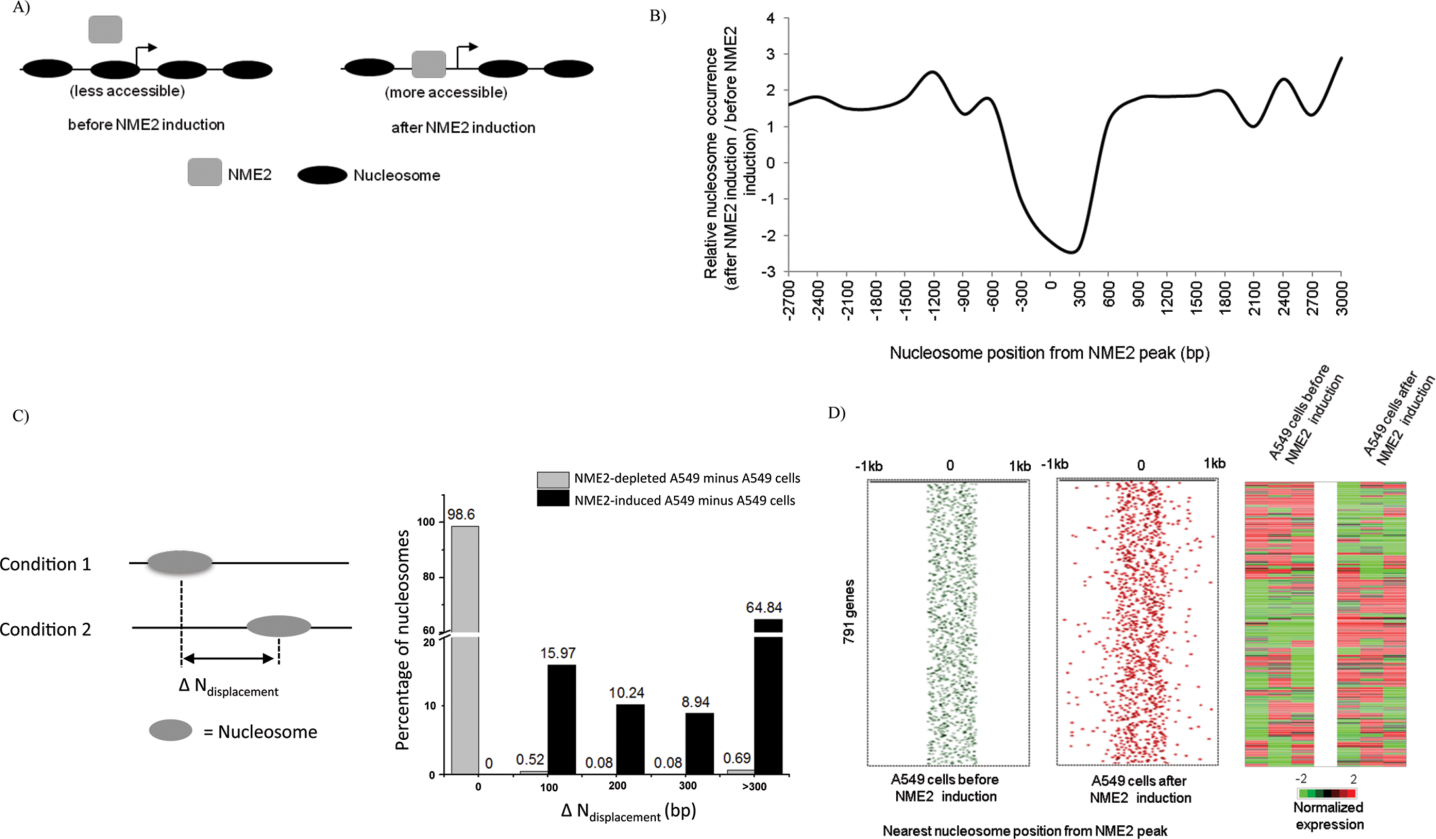
Nucleosome depletion and NME2 occupancy. (**A**) Schematic representation of possible relationship between nucleosome positions before and after inducing NME2. (**B**) Nucleosome occupancy is depleted on or near NME2 binding sites on inducing NME2 relative to the condition before NME2 induction. Ratio of number of nucleosomes detected after/before NME2 induction in 300 bp windows is shown; x-axis denotes the distance of nucleosomes from the nearest NME2 binding site in NME2-induced cells. (**C**) Schematic representation of nucleosome shift between two conditions was represented by Δ*N*_displacement_ (left panel). Percentage of shifted nucleosomes plotted for a given Δ*N*_displacement_ is shown in the right panel (x-axis was plotted to indicate: no shift, shift in 100 bp windows and shift exceeding 300 bases). Distribution of the nucleosome shift was also found for the NME2-depleted condition minus A549 cells; significance of the difference in distributions was tested using the Wilcoxon rank sum test (*P* = 0.00016). (D) Position of the nearest nucleosome with respect to NME2 binding sites in cells before (green) or after inducing NME2 (red; left panels); 791 genes where the nearest nucleosome was within 300 bp and shifted in the NME2-induced condition are shown. Expression level of corresponding genes in triplicate before or after NME2 induction is shown in the right panels.

**Figure 4.**
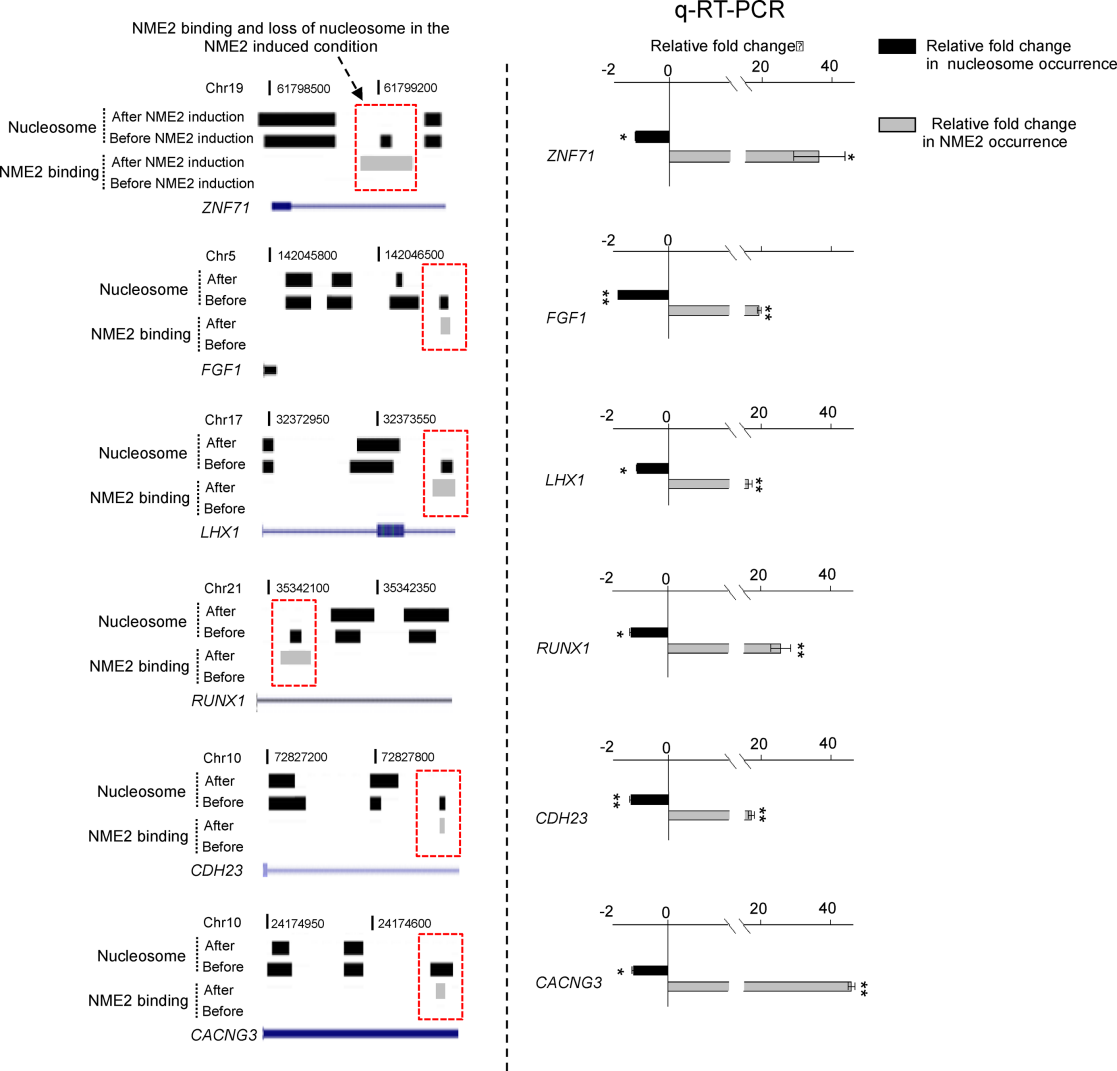
Validation by quantitative real-time PCR (Polymerase Chain Reaction) for NME2 binding and nucleosome occupancy. Nucleosome positions and NME2 binding sites are shown before and after NME2 induction at six different loci in the left panel (genes shown in UCSC browser representation); right panel shows validation at the corresponding loci by quantitative real-time PCR for NME2 binding and nucleosome occupancy in cells before and after inducing NME2. Relative fold change is shown on x-axis. Experiments were performed in triplicate; error bars are for standard deviation (* and ** represent *P* < 0.05 and *P* < 0.01, respectively).

**Figure 5.**
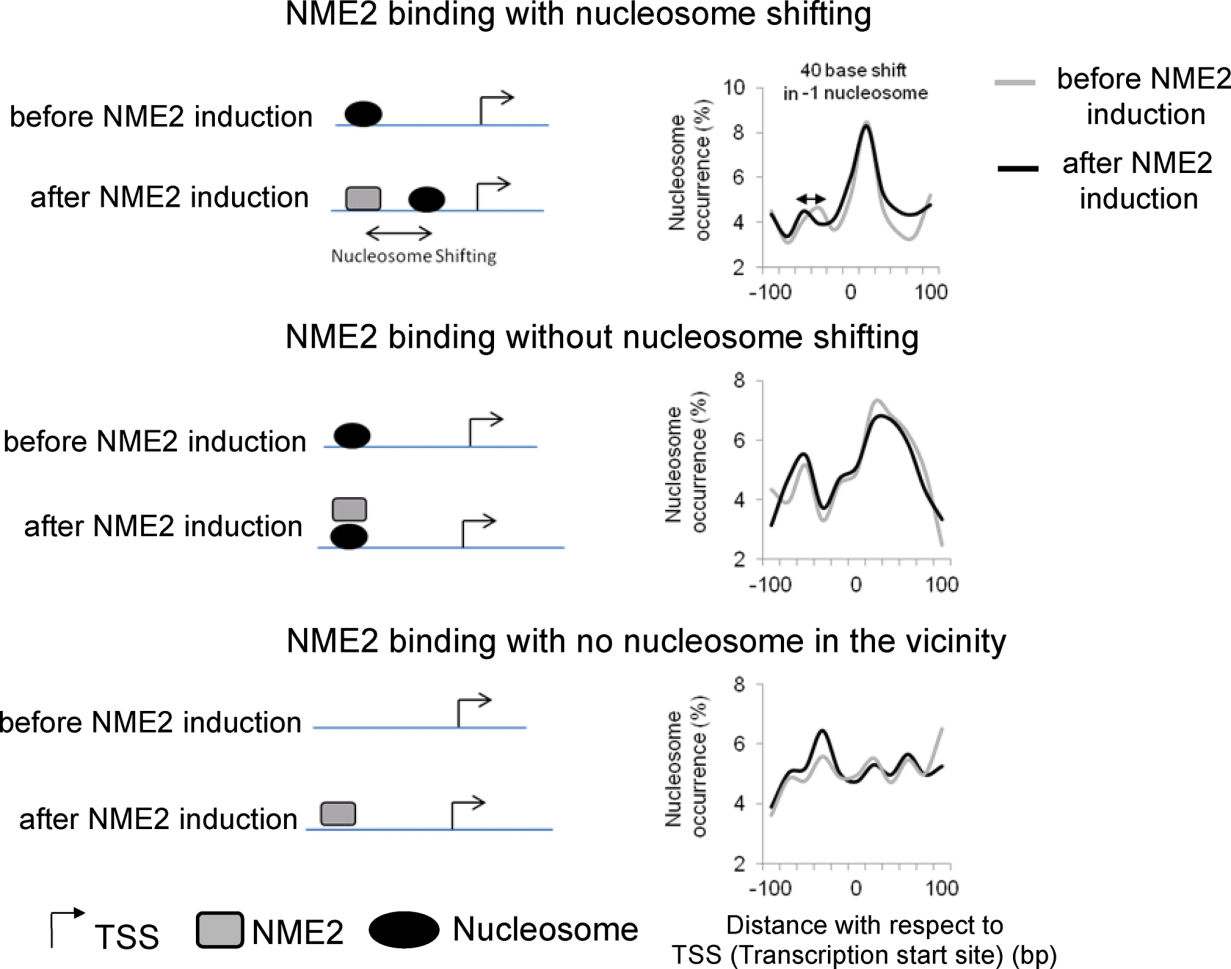
TF binding and nucleosome repositioning models analyzed in this study. Nucleosome shift in the vicinity of TF binding results in repositioning of the −1 nucleosome (upper panel), whereas co-occupancy (center panel) or target site binding that has no associated nucleosome repositioning in close vicinity either before or after induction of the TF (lower panel) shows no significant change in −1 or +1 nucleosome positions. Percentage of nucleosomes is based on genome-wide total for respective cases; to avoid arbitrary assignment genes were considered in more than one category where applicable.

### Quantitative real-time PCR for validating ChIP-nucleosome positioning

For validating individual nucleosome positions, quantitative real-time PCRs were performed using ChIPped DNA on ABI's (Applied Biosystems, Inc.) 7500 fast platform. Equal concentration of ChIP DNA was taken from the test (from NME2-induced cells) and contro‘l (un-induced) samples. The results were analyzed using comparative computed tomography method. Primers were designed against the obtained peak region using ABI's Primer Express software (Supplementary Table S1).

### Western blotting

Western blot for NME2 was done using antibody from Abcam, Cambridge, MA, USA (catalog no. ab60602). Actin was probed by antibody from Sigma (catalog no. 1978).

The gene expression, nucleosome positioning and ChIP-seq data have been submitted to Gene Expression Omnibus (http:/www.ncbi.nlm.nih.gov/geo/) under accession numbers GSE40194, GSE40300, GSE40363 and GSE18182.

## RESULTS

We analyzed expression of 1722 regulatory factors, including ones involved in cancer metastasis (27–29), in transcriptome profiles of 382 lung cancer clinical cases. Our analyses identified that a metastasis suppressor NME2 (also known as NM23 H2/NDPK) was significantly down regulated in advanced stages across four independent data sets (*P* < 0.05, student's *t*-test; Supplementary Figure S1a). Details of this analysis including further experiments performed by us which confirmed that NME2 can induce anti-metastatic changes in lung adenocarcinoma-derived A549 cells are provided as supplementary material (Supplementary Information and Supplementary Figures S1b–d). Next, we determined NME2 occupancy, nucleosome positions and transcriptomes before and after induction of NME2 levels in A549 cells. As NME2 is endogenously expressed in A549 cells, we used the term ‘NME2 induced’ to refer to the cellular state following increase of NME2 levels. To ascertain whether NME2 protein level after induction in A549 cells was still in the physiological range, we analyzed protein expression of NME2 in normal lung lysates and tumor lung, and compared with NME2-induced A549 cells. Our comparative analysis clearly showed that NME2 level within induced A549 cells were within physiological range (Supplementary Figure S1e).

### Genome-wide NME2 binding, promoter-nucleosome occupancy and the transcriptional state

ChIP followed by massively parallel sequencing (ChIP-seq) was performed in replicate for both conditions, before and after inducing NME2 (Figure 1A). Out of roughly 7 million sequence reads, >80% uniquely aligned to the reference human genome in each case (Supplementary Figure S2a). Using ChIP-seq reads enriched in NME2 relative to IgG, we found 2005 and 11 017 peaks before and after inducing NME2 in A549 cells, respectively. Although, number of NME2 binding sites increased after induction, a close inspection of peaks (sites of NME2 binding to DNA) showed that their chromosome-wise distribution was largely similar in both cases (Figure 1A: Circos plot, and Supplementary Figures S2b and c). The replicates were also similar (Figure 1A: Circos plot, and Supplementary Figure S2d). The frequency of TFBSs was also largely similar before and after induction of NME2 (Supplementary Figure S2e). A 12-mer consensus motif identified using Gibbs sampler (30) was present in >70% of the ChIP-seq peaks (Figure 1B upper panel). We noted that the motif found from ChIP-seq was similar to the one reported earlier from analysis of a single promoter (31) and unpublished data of Thakur *et al*. (submitted for publication) As expected, in more than 50% cases the NME2 motif occurred within 50 bp of the center of peak (Figure 1B lower panel). We next performed transcriptome profiling of A549 cells before and after NME2 induction. Comparative analysis of the expression profiles revealed 1679 genes as differentially expressed (781 genes were up and 898 genes down regulated (*P* < 0.05)) in NME2-induced cells; out of these we found at least one NME2 binding site (in the induced condition) within 10 kb of the TSS in as many as 1235 genes.

We next asked whether altered state of chromatin in regulatory regions influenced NME2 occupancy and consequent gene expression changes. To test this, nucleosome positions were determined in the A549 cells before and after NME2 induction. Mono-nucleosomes were isolated by MNase digestion and we mapped nucleosome positions to putative promoter regions (−7.5 kb to 2.5 kb of TSSs) using tiled microarrays (Figure 2A) and found 157 634 and 162 570 nucleosomes before and after NME2 induction, respectively. Next, we checked the relationship between overall number of nucleosomes on each promoter and the expression level of corresponding gene and found that enriched promoter-nucleosome occupancy correlated with decreased expression of corresponding genes (*r* = −0.73; *P* < 0.05 before NME2 induction and *r* = −0.80; *P* < 0.05 after NME2 induction, Pearson correlation; Figure 2B).

### Target site proximal promoter-nucleosome repositioning upon NME2 expression

We noted distinct occurrence of nucleosomes with respect to TSSs across promoters for many genes, which showed a phased pattern that was consistent with previous studies of nucleosome distribution in human and yeast (15,32,33) (Figure 2C). On comparing the two states, before and after NME2 induction, we found ∼11.4% (18 024/157 634) nucleosomes on 1022 genes were repositioned in the NME2-induced condition. We also found that a large number of genes with repositioned nucleosomes were differentially expressed between the two states (830 out of 1022 genes (*P* < 0.05)) (Figure 2D). It is important to note that in contrast to Figure 2B which showed that a high overall increased number of nucleosomes on a given promoter relates to decreased expression, Figure 2D showed that differential expression of genes here resulted from repositioning (and not overall exclusion or gain) of nucleosomes within promoter. Interestingly, most repositioning events after NME2 induction were observed near TSSs (Figure 2D, upper panel).

We next checked target-site nucleosome occupancy before and after NME2 induction (Figure 3A). On analyzing relative occurrence we found lower number of positioned nucleosomes in the vicinity (∼300–500 bp) of NME2 target sites in cells after NME2 induction (Figure 3B). Out of 3956 NME2 target sites (within −7.5 to +2.5 kb of TSS) unique to the NME2-induced cells, 1257 (31%) present on 1119 putative promoters were found to either overlap or were within 300 bases of a nucleosome in cells before NME2 induction. Furthermore, 870 (∼70%) of the 1257 sites were found to be nucleosome-free in the NME2-induced condition, which involved repositioning of 870 nucleosomes in 791 genes on NME2 induction. Together, these findings indicate that many of the NME2 binding sites occupied by nucleosomes in the un-induced condition in A549 cells became NME2-bound (and nucleosome-free) in the NME2-induced condition.

To analyze nucleosome repositioning with respect to the NME2 target site after versus before NME2 induction, we calculated the distance of nucleosome shift between the two conditions as Δ*N*_displacement_ (Figure 3C, left panel) and found ∼64% nucleosomes shifted by more than 300 bp in the NME2-induced condition. In order to test the significance of the noted nucleosome shift, we also calculated this distribution in A549 cells where NME2 was depleted (see below) relative to control A549 cells. Observed distributions were significantly altered following induction of NME2; in contrast, on NME2 depletion we did not find any repositioning in most cases (Wilcoxon rank sum test; *P* = 0.00016; Figure 3C, right panel). Next we plotted the 870-nucleosome positions (found within 300 bp of an NME2 target site in the un-induced condition) before and after NME2 induction. This showed a loss in organized nucleosome occurrence around NME2 binding sites in NME2-induced cells relative to the un-induced condition (Figure 3D). We further noted that expression of all the 791 genes with repositioned nucleosomes was significantly altered in the NME2-induced condition (*P* < 0.05, student's *t*-test, Figure 3D). For validation, we compared nucleosome occupancy and NME2 binding using quantitative real-time PCR in six genes. In all cases, low nucleosome occupancy signal was detected along with high NME2 occupancy in cells after NME2 induction as compared to that observed in un-induced cells (Figure 4).

### Target sites are nucleosome occupied in cells with depleted levels of NME2

We reasoned that in addition to induced NME2; A549 cells with depleted levels of NME2 would provide a suitable model to test nucleosome positioning/NME2 occupancy in a contrasting situation. To test this, we generated a stable NME2-depleted A549 cell line (see Materials and Methods). Following this we determined nucleosome positions in the NME2-depleted cells. All the 870 NME2 binding sites that were nucleosome-free in the NME2-induced condition and occupied in the un-induced state (Figure 3D) were also found to be nucleosome-occupied in the NME2-depleted condition. To further functionally validate this point, we show two promoters where the NME2 target site was not occupied by NME2 and had positioned nucleosomes in A549 cells and NME2-depleted A549 cells but were available for NME2 binding following NME2 induction (Supplementary Figure S3). In case of 53 genes, we found positioned nucleosomes on or near NME2 target sites on NME2 depletion relative to control A549 cells. Together this suggests that in contrast to the nucleosomal changes following increase in NME2 expression, NME2 target sites remain nucleosome-occupied in most cases on depletion of NME2.

### Binding site occupancy is transcriptionally active when associated with nucleosome repositioning

We found that occupancy of about a fifth (870 of 3956 NME2 target sites, ∼22%) of the transcription target sites was concurrent with repositioning of nucleosomes in the NME2-induced condition. Interestingly, these repositioning events resulted in altered expression of all the 791 genes (Figure 3D). In contrast, we found 1175 genes where the NME2 binding site (unique to the induced condition) was co-occupied with nucleosomes—only 130 (11%) of these genes showed altered expression. As a third possibility, we found 1990 genes with NME2 occupancy in the induced condition though no nucleosomes were present in the vicinity of the NME2 site either before or after induction—i.e. target sites appeared to be independent of nucleosome repositioning. Again, out of 1990, only 179 (8.9%) genes were differentially expressed. On mapping the NFR between the −1 and +1 nucleosome positions in each of the three situations described above, we found repositioning of the −1 nucleosome by ∼40 bp in the first case when repositioning was linked to binding site occupancy, whereas in the other two situations the NFR was minimally altered on inducing NME2 (Figure 5).

## DISCUSSION

Our findings suggest that TF binding when closely associated with nucleosome repositioning results in altered gene expression changes. Interestingly, in most cases when TF binding did not impact local nucleosome reorganization it was not associated with altered transcriptional state of target gene. As we used human cancer cells that are metastatic, and expression of the TF NME2 decreased their metastatic potential, these findings also help in understanding how TF binding-induced nucleosome level changes influence the transcriptome during metastasis.

### TF binding and transcriptional activity are linked through local nucleosome repositioning

It was recently reported that repositioning of the +1 nucleosome resulted in changes to NFR in genes that were differentially regulated during meiotic development in yeast (9). Though this was noted as a result of change in possibly multiple regulatory factors involved in meiotic development, it is consistent with our results. Furthermore, our findings indicate that assignment of transcriptional function to genome-wide target site binding would require information on nucleosome reorganization to be more precise. This helps explain the noted discrepancy in high throughput DNA binding studies where low overlap between experimentally determined binding sites and gene expression has been observed (34,35). A recent study noted chromatin accessibility before and after binding of the receptors (androgen (AR) or estrogen (ESR1)) were significantly altered (19) and suggested that both AR and ESR1 binding are associated with changes in local nucleosome occupancy. This is in line with our findings and suggests a model that integrates factor binding and transcriptional activity of genes with local nucleosomal changes.

Non-specific binding of NME2 in the induced condition could be a confounding factor. To address this, first, we checked and found that in NME2-depleted cells a large number of genes were oppositely expressed with respect to their status in NME2-induced cells; it is unlikely that non-specifically activated/repressed genes as a result of NME2 induction would be differentially expressed on depleting NME2 (Supplementary Figure S4). Second, the differentially expressed genes in NME2-induced cells correlate significantly with transcriptome changes that are clinically relevant (Supplementary Figure S5). Therefore, though all NME2 binding events do not lead to increase/decrease in transcription it is unlikely to be due to spurious binding—it is possible that many of these associations are required for functions other than transcription.

### Overall chromatin landscape in promoters is largely constant, site-specific changes are associated with transcription

We found only 11.4% of nucleosomes to be repositioned in promoter proximal regions as a result of NME2 induction. Therefore, it is interesting to consider that overall chromatin level changes may be relatively small. On the other hand, and perhaps more interestingly, there may be shift in nucleosome occupancy on TF binding, leading to site-specific ‘open’ or ‘closed’ regions that facilitate regulatory events. Our findings (discussed above) further support this: nucleosomes repositioning along with engagement of TF at specific sites were in almost all cases associated with transcriptional change in the corresponding gene. In addition, in both cases before and after NME2 induction, enriched promoter nucleosome occupancy correlated with decreased expression of genes. Together these support a model where nucleosome occupancy generally determines the suppressed state of the transcriptome, and reorganization induced by DNA binding factors (themselves or when associated with chromatin modifiers) results in transcriptional activation at specific loci. Although further studies using other TFs will be required to substantiate this, it appears to be consistent with an earlier study which observed decreased presence of nucleosomes in promoters of genes that were expressed during heat shock in yeast (16). However, others have also noted either unchanged nucleosome occupancy (yeast grown in different carbon sources (36,37)) or found nucleosome positioning to correlate with the state of transcription (active or silent), and not the extent of gene expression (18).

Epigenetic signaling directs the location of TFs to cognate sites in given chromatin territories. Following this, TFs are believed to be one of the key recruiters of chromatin modification and remodeling machineries (38–40). Recent evidence suggests that even the general TFs, such as subunit of TFIID (Transcription Factor II D) complexes, may be functional component of these machineries (41). In agreement with this basic understanding of transcription through chromatin, our results demonstrate TF binding to be transcriptionally competent when coupled with locally altered nucleosome positioning. Furthermore, our findings for the first time underline the importance of these aspects of chromatin biology in suppression of cancer spread mediated by NME2. However, it remains to be elucidated whether NME2-mediated alteration of nucleosomal reorganization possesses any unique features of the histone modification language (involving specific enzyme complexes and histone chaperones) that could be essential in mitigating metastasis.

## SUPPLEMENTARY DATA

Supplementary Data are available at NAR Online.

SUPPLEMENTARY DATA
