# Supplementary material for: Promoter-proximal transcription factor binding is transcriptionally active when coupled with nucleosome repositioning in immediate vicinity
Source: Nucleic Acids Res. 2014 Jul 31;42(15):9602–11. doi: 10.1093/nar/gku596 (PMC4150765; doi:10.1093/nar/gku596)
Supplement: SUPPLEMENTARY DATA [file supp_42_15_9602__index.html]

Promoter-proximal transcription factor binding is transcriptionally active when coupled with nucleosome repositioning in immediate vicinity — SUPPLEMENTARY DATA 

# Promoter-proximal transcription factor binding is transcriptionally active when coupled with nucleosome repositioning in immediate vicinity

## SUPPLEMENTARY DATA

**Files in this Data Supplement:**

- SUPPLEMENTARY DATA
