# Supplementary material for: Promoter-proximal transcription factor binding is transcriptionally active when coupled with nucleosome repositioning in immediate vicinity
Source: Nucleic Acids Res. 2014 Jul 31;42(15):9602–11. doi: 10.1093/nar/gku596 (PMC4150765; doi:10.1093/nar/gku596)
Supplement: SUPPLEMENTARY DATA [file supp_gku596_nar-03354-x-2013-File002.docx]

**Supplementary information**

**Yadav et al., 2013**

**Analysis of clinical transcriptomes**

Transcriptome profiles of 382 lung adenocarcinoma clinical cases (from four independent studies, references in manuscript text) were screened for 1722 regulatory factors. Each transcriptome was grouped as advanced (stage III and IV) or early (stage I and II); all pathological stage information was used as provided by the individual studies or as certified by the pathology report from the expO (International Genomics Consortium database where gene expression data from tissue samples procured and clinically annotated under standard conditions is publicly available ([www.intgen.org/expo](http://www.intgen.org/expo))). Gene expressions across datasets were normalized using the Z score transformation as reported earlier(42), differential expression of 1722 regulatory factors was tested between the two groups (early or advanced) and statistical significance analyzed using the student’s t-test. Twenty-three factors were found to be significantly differentially expressed in at least two of the four studies analyzed (*P*<0.05; Supplementary Figure 1a). As expected, many of the 23 factors identified by this analysis have been previously implicated in cancer progression across cancer types; notably FOSL2, FOXD3 and HSF1 in metastasis of breast cancer, melanoma and hepatocellular carcinoma respectively (43-45). NME2 was selected for further work as it was significantly repressed in advanced stages in all four independent studies.

**Analysis of NME2 levels and patient survival data**

Survival data from four independent previous studies were analyzed for correlation with NME2 transcript levels. Normalized values of NME2 expression were used where available, in cases, where only raw signal intensity was given normalization was done after z-transformation as mentioned above. We analyzed correlation of individual NME2 levels and survival for each patient using Pearson correlation (Supplementary Figure 1b).

**Comparative analysis of gene expression patterns between clinical sample datasets and NME2-induced/depleted A549 cells**

In order to test the overall correlation between gene expression difference observed in clinical samples versus the changes observed on depleting or inducing NME2, we first pre-processed the datasets using a previously published method(46). Briefly, for clinical samples, v (defined as relative fold change for each gene) between expression of advanced versus early stages was calculated using

v = s (1− p)

where ‘p’ is the p-value calculated by performing a student’s t-test for expression difference between the stages of cancer samples, ‘s’ denotes the sign of the difference between the average values in the two sets considered. Therefore, ‘v’ indicates the extent to which a gene was up or down-regulated in the advanced stage with maximal and minimal values of 1 and -1 respectively. ‘v’ for genes from the dataset of NME2-induced A549 cells was calculated similarly using respective control cells. Following this, ‘v’ obtained from the clinical samples was compared with that from NME2-induced condition for respective genes; statistical significance of this comparison was tested using the Pearson correlation test.

**Gene set enrichment analysis (GSEA)**

GSEA analysis was done as reported earlier (47). Briefly, a set of differentiallyexpressed genes (in NME2-induced state relative to the un-induced condition) was given as the query set. Each query gene set was checked for enrichment within the list of genes ordered on the basis of expression difference between the two diagnostic classes (early or advanced stage of lung cancer). Enrichment score (ES) based on Kolmogorov-Smirnov (K-S) running sum statistic was calculated for each query set. To determine whether a query gene set was enriched in one diagnostic class relative to the other, the entire procedure of ES calculation was repeated 1,000 times, using permuted diagnostic assignments. This provides a global P value for assessing whether any gene set was associated with the diagnostic categorization at a significance cut off (*P<* 0.05; FDR < 5%).

Analysis of >47,000 transcripts following NME2 induction revealed 781 genes to be up and 898 genes as down regulated (*P*<0.005). GSEA with the profile obtained from 93 lung cancer samples (after subtracting the profile of early from advanced samples) showed that genes repressedin NME2-induced cells were down regulated in early lung cancer samples (Supplementary Figure 1e; left panel; NES=-1.5; *P*=0.03) and similarly, up regulated genes in A549 cells had increased levels in theearly stages (Supplementary Figure 1e; right panel; NES=1.25; *P*=0.04).

**Supplementary Figure Legends**

**Supplementary Figure 1. (a)** Left panel:Analysis of differential expression of 1722 regulatory factors between early (stages 1&2) and advanced (stages 3&4) of lung cancer in four independent clinical transcriptome profiles; expression index: red, up-regulation and blue, down-regulation. Right panel:heat map for 23 regulatory factors that showed reduced expression in advanced tumor stages in at least two data sets; expression index: red, significant down regulation and black, no change. **(b)** Correlationbetween patient survival and NME2 expression in individual cases for the all four studies; normalized NME2 expression, as available from individual studies or z-transformed by us in cases where raw intensity was given; statistical significance was tested using Pearson correlation. **(c)** NME2 expression, mRNAof A549 cells following treatment with NME2-specific siRNA or control si-RNA. **(d)** Gene Set Enrichment Analysis (GSEA) of two gene sets representing NME2-repressed (left) and NME2-activated (right) genes in 93 lung cancer samples grouped as early versus advanced stage (see Supplementary Information). NES, normalized enrichment score; p = nominal p value; q value = corrected p-value; heatmap on the bottom of each panel represents the ranked, ordered, non-redundant list of genes - on the far left (red) up-regulated in early stage samples, and genes on the far right (blue) up-regulated in advanced stage samples. On each panel, the vertical black lines indicate position of genes of the queried gene set (NME2 repressed or activated) in the ordered, non-redundant data set (early versus advanced stage lung cancer). The green curve corresponds to the ES (enrichment score) curve, which is the running sum of the weighted enrichment score obtained using the GSEA algorithm. (e) Comparison of NME2 level across cell lines, normal lung and lung tumor sample; the upper panels represent western blots to check NME2 protein expression across different types of samples; actin was used as loading control. The lower panels represent spot densitometric analysis of bands; expression in A549 is given unit value and subsequently used for comparison.

**Supplementary Figure 2. (a)** ChIP-seq reads for NME2. Reads were mapped on human reference genome and uniquely mapped reads were retained for further analysis; percentage of uniquely aligned reads was >80% in most cases. **(b)** Distribution of NME2 binding sites before and after induction: across different human chromosomes; **(c)** distribution of NME2 peaks with respect to genomic locations; TSS (transcription start site), within gene and downstream of TES (transcription end site). **(d)** fold enrichment analysis of peaks across replicates; x and y axes represent fold enrichment of a peak relative to input control (2-6 fold represented here); N= number of peaks; each solid circle represents a peak; R represents correlation coefficient. **(e)** Transcription factor binding sites (TFBS) enriched within NME2 peaks in cells before (left panel) or after (right panel) induction of NME2.

**Supplementary Figure 3.** Representative examples of two promoters where NME2 binding was excluded (due to nucleosome positioning) in cells before NME2 induction or on NME2 depletion and were available on inducing NME2.

**Supplementary Figure 4.** Expression of 1051 genes in NME2 induced condition were reversed on depletion of NME2 in A549 cells (P<0.05, student’s t-test).

**Supplementary Figure 5. (a)** Expression signature of 450 genes differentially expressed after inducing NME2 in A549 cells that are significantly correlated with expression change found in early lung cancers relative to advanced stages in two independent clinical studies;**(b)** Pearsoncorrelation of relative fold change in expression between clinical samples and NME2 induced A549 cells.

**Supplementary Figure 1**

(b)


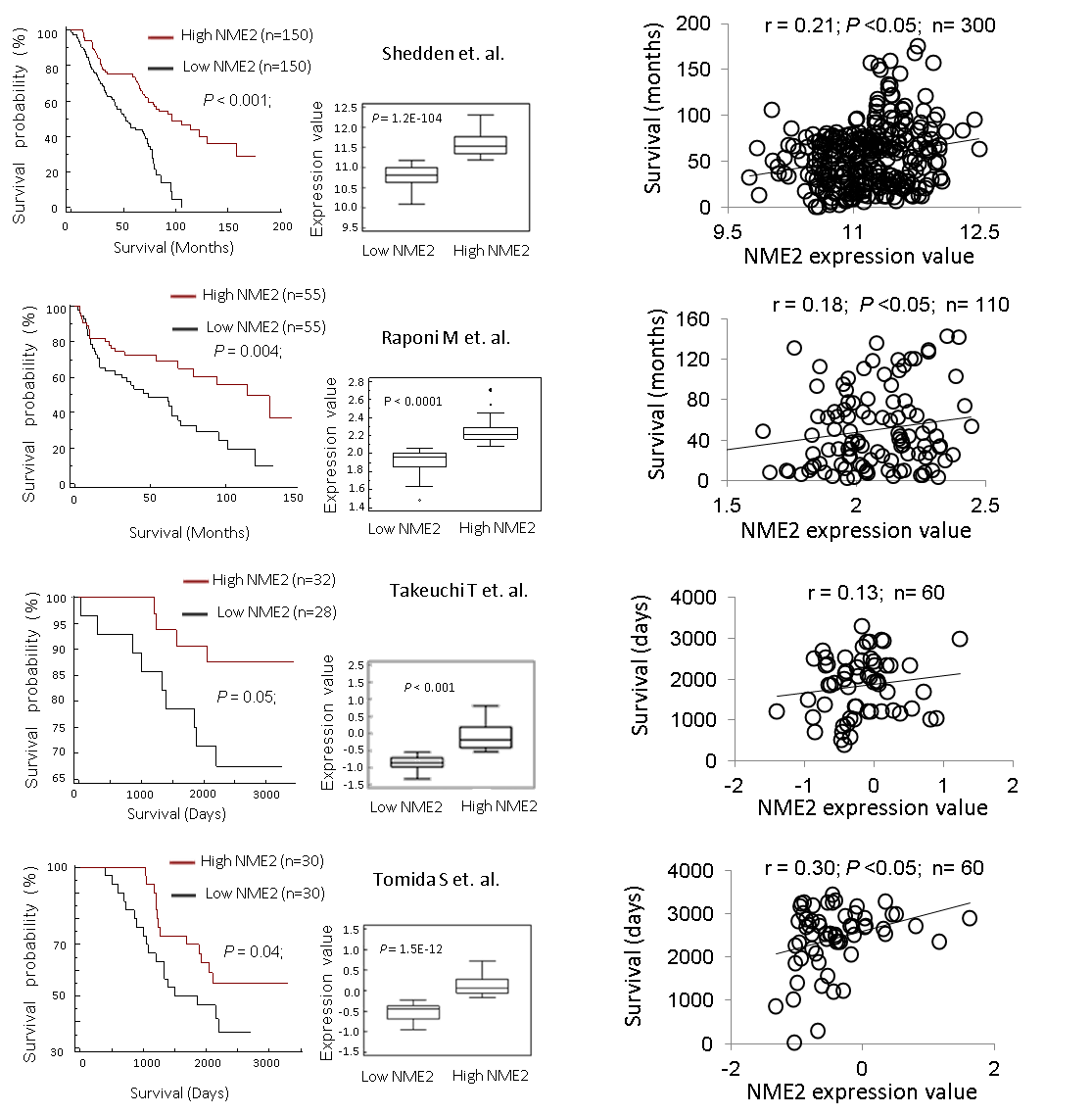


(c)


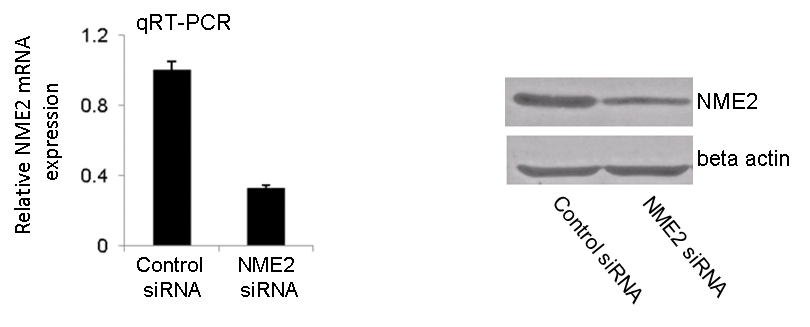


(d)

**
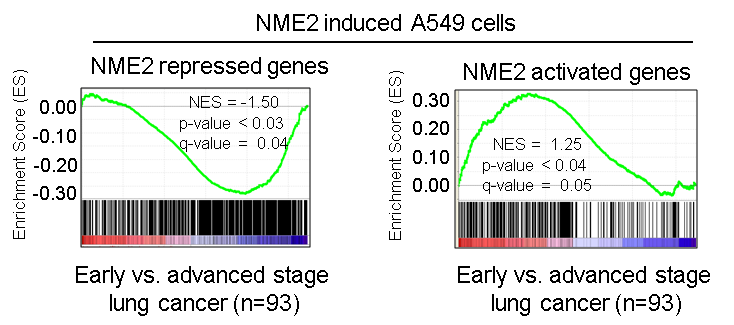
**

**(e)**

**
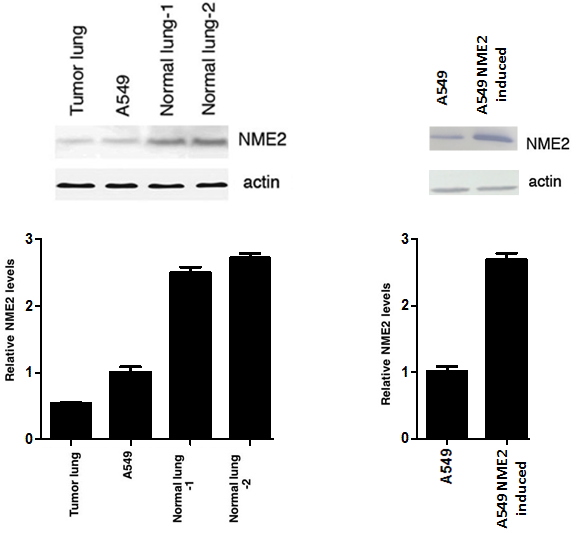
**

**Supplementary Figure 2**

(a)


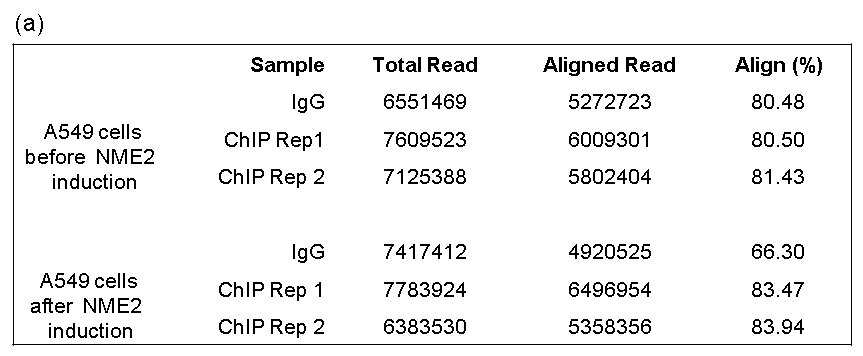


(b)

(c)


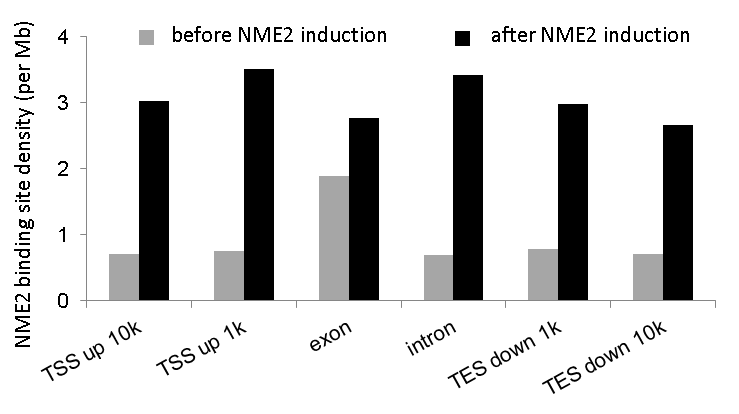


(d)


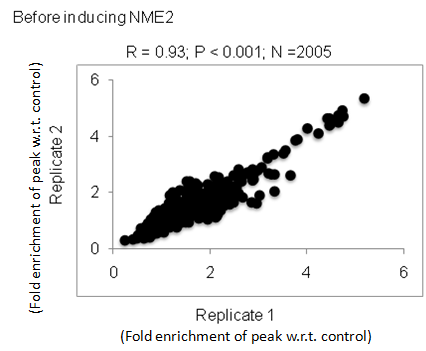


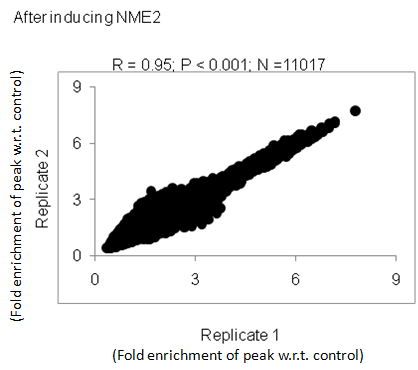


(e)


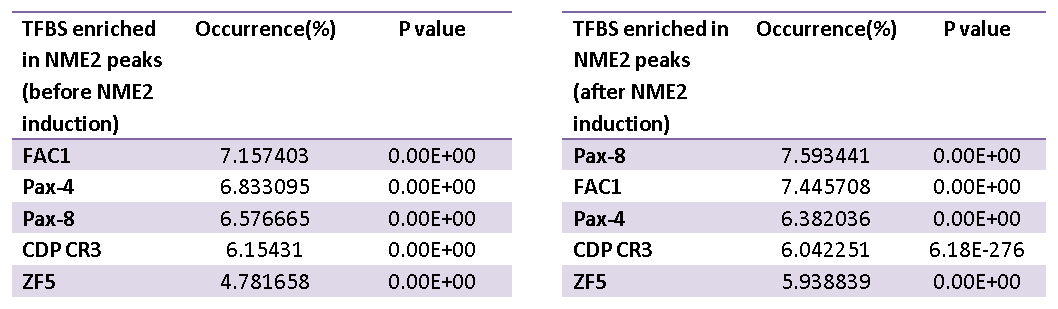


**Supplementary Figure 3**


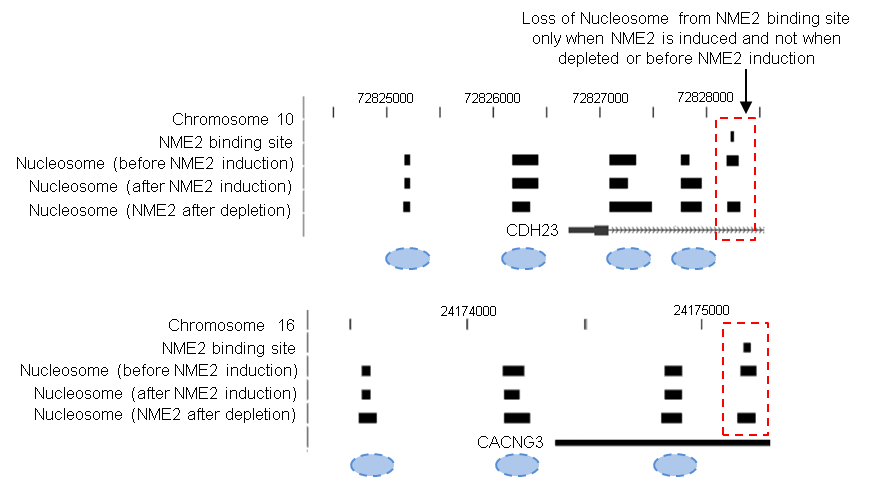


**Supplementary Figure 4**


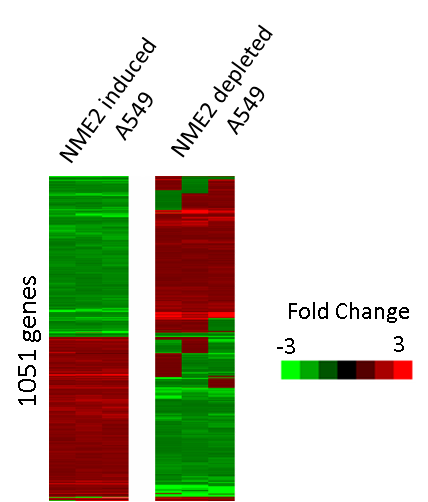


**Supplementary Figure 5**

(a)

(b)

**
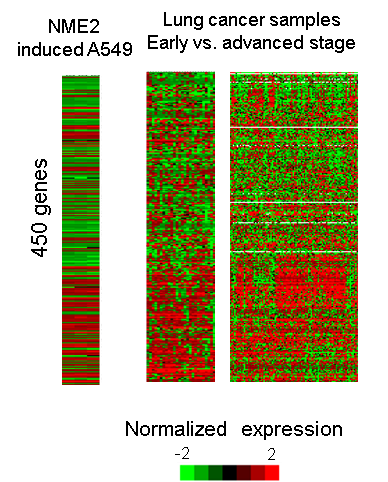

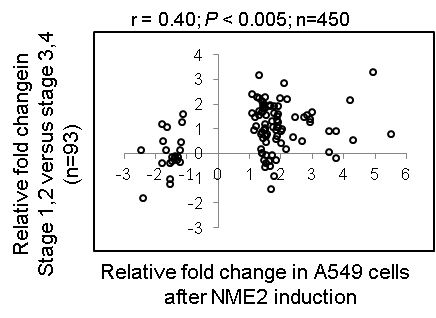
**

**Supplementary Table 1**

**Primer information for validation of results from nucleosome and NME2 binding sites**

|  | forward primer | reverseprimer |
| --- | --- | --- |
| ZNF71 | TGGAGGTAGGAAAGTTGCATGA | ATGCACTGCCTGTCTCTCACA |
| FGF1 | ACACAAGCCTGCTCACCTGAT | CTGGAGAGCATATTTCTAGTTCTGACA |
| LHX1 | CGACTATGAGAGCGCTGTTTGT | TCTCCTGCGCCCAATGAG |
| RUNX1 | TCCCAGCCCACTCCATCTC | CCAGTGCCTGCAAAGTGTATCT |
| CDH23 | GATGATGAGTCAGCCCACGAT | CCCTGCACCACGAACCA |
| CACNG3 | GAGCAGGCGGAGAGCTTTC | GCGAGCCGCTGAGAAGAGT |

Reference List

42. Cheadle, C., Vawter, M.P., Freed, W.J. and Becker, K.G. (2003) Analysis of microarray data using Z score transformation. *J.Mol.Diagn.*, **5**, 73-81.

43. Fang, F., Chang, R. and Yang, L. (2012) Heat shock factor 1 promotes invasion and metastasis of hepatocellular carcinoma in vitro and in vivo. *Cancer*, **118**, 1782-1794.

44. Katiyar, P. and Aplin, A.E. (2011) FOXD3 regulates migration properties and Rnd3 expression in melanoma cells. *Mol.Cancer Res.*, **9**, 545-552.

45. Milde-Langosch, K., Janke, S., Wagner, I., Schroder, C., Streichert, T., Bamberger, A.M., Janicke, F. and Loning, T. (2008) Role of Fra-2 in breast cancer: influence on tumor cell invasion and motility. *Breast Cancer Res.Treat.*, **107**, 337-347.

46. Goodarzi, H., Elemento, O. and Tavazoie, S. (2009) Revealing global regulatory perturbations across human cancers. *Mol.Cell*, **36**, 900-911.

47. Subramanian, A., Tamayo, P., Mootha, V.K., Mukherjee, S., Ebert, B.L., Gillette, M.A., Paulovich, A., Pomeroy, S.L., Golub, T.R., Lander, E.S. *et al.* (2005) Gene set enrichment analysis: a knowledge-based approach for interpreting genome-wide expression profiles. *Proc.Natl.Acad.Sci.U.S.A*, **102**, 15545-15550.
